# Parents’ subjective understanding, use, reasons for non-use and perceived effectiveness of food labelling formats in Germany: a cross-sectional study of the Nutri-score, nutrition information panel and ingredients list

**DOI:** 10.1186/s12889-026-29571-8

**Published:** 2026-09-29

**Authors:** Anja Simmet, Janine Ehret, Nana Ama Frimpomaa Agyapong, Nanette Stroebele-Benschop

**Affiliations:** 1 Institute of Clinical Nutrition, Department of Applied Nutritional Psychology, University of Hohenheim, Fruwirthstr. 12, Stuttgart, 70599 Germany; 2 Department of Nutrition and Dietetics, School of Allied Health Sciences, College of Health and Allied Sciences, University of Cape Coast, Cape Coast, Ghana

**Keywords:** Food labelling, Front-of-pack labelling, Nutri-score, Nutrition information panel, Parents, Consumer behaviour

## Abstract

**Background:**

Food labels are intended to support healthier food choices, yet little is known about how parents engage with different labelling formats in Germany. This study examined parents’ subjective understanding and use of three food labelling formats in Germany, the Nutri-Score, the Nutrition Information Panel (NIP) and the list of ingredients and identified factors associated with subjective understanding and use, reasons for non-use and perceptions of label effectiveness.

**Methods:**

Cross-sectional data were obtained from an online survey of parents in Germany (*n* = 978). Participants were adults responsible for at least 50% of household food shopping, who lived with children under 18 years. Quota-based sampling approximated the distribution of parents by household income and municipality size. Descriptive analyses were used to summarize subjective understanding, use, reasons for non-use and evaluative aspects of the three food labelling formats. Multivariable logistic regression analyses were conducted to identify characteristics associated with subjective understanding and use, and generalized estimating equations were used to compare evaluative aspects across the three food labelling formats.

**Results:**

Most parents reported subjective understanding of the Nutri-Score (84.8%), the NIP (83.7%) and the ingredients list (80.5%) and 73.7% reported subjective understanding of all three formats. Overall, 70.3% used the Nutri-Score, 74.2% the NIP and 70.6% the ingredients list, while 58.8% reported using all three. Stronger beliefs in the effectiveness of healthy eating were positively associated with subjective understanding and use, whereas higher perceived overload from nutrition information was negatively associated. Men were less likely than women to report subjective understanding or use of the NIP and the ingredients list. Lack of interest was the most frequently reported reason for non-use. More participants agreed that the Nutri-Score was easy to use, whereas more agreed they understood the information provided by the NIP.

**Conclusion:**

A high proportion of German parents in this quota-based sample reported subjective understanding and use of multiple food labelling formats. Subjective understanding and use were consistently associated with beliefs about the effectiveness of healthy eating and perceived nutrition information overload. These findings may help inform future public health communication on food labelling.

**Clinical trial number:**

Not applicable.

## Background

Adherence to a diet rich in fruits, vegetables and whole grains and low in free sugars, salt and saturated fats is strongly associated with reduced disease risk and improved health outcomes throughout the life course [1]. Dietary habits established during childhood are particularly important, as they often persist into adulthood [2], and poor childhood diet quality has been linked to increased cardiometabolic risk later in life [3]. Because parents largely determine the foods available and consumed within the household, they play a central role in shaping these habits through food purchasing decisions, meal preparation and the home food environment [4].

This is of particular public health relevance because poor dietary quality, regardless of age group, constitutes a leading modifiable risk factor for non-communicable diseases (NCDs) such as obesity, cardiovascular disease, type 2 diabetes and certain cancers, and is a major contributor to morbidity, premature mortality and disability-adjusted life years (DALYs) [5]. In Germany, obesity alone accounts for an estimated 102 000 premature deaths annually and represents a considerable economic burden. In 2015, direct healthcare costs attributable to obesity were estimated at €23.39 billion, with an additional €33.65 billion in indirect costs [6]. Promoting healthy dietary behaviour in general and among parents in particular is therefore a central component of public health strategies aimed at preventing NCDs and reducing healthcare costs [7].

Food labelling is widely regarded as an important policy tool to support healthier food choices by providing consumers with accessible nutrition information. In the European Union (EU), packaged foods are required to display a nutrition information panel (NIP), which provides detailed quantitative information on nutrient composition such as energy, fat, carbohydrates, sugars, protein and salt [8]. In addition, EU legislation mandates that prepackaged foods include a list of ingredients in descending order by weight, with allergens clearly indicated [8, 9]. These mandatory labelling formats serve distinct but complementary purposes. While the NIP enables consumers to evaluate products based on their nutrient composition [8], the ingredients list increases transparency regarding product composition and can support consumers in identifying allergens, added sugars, additives, or other ingredients relevant to their dietary preferences and health needs [10]. However, interpreting this information often requires time, motivation and nutritional knowledge, which may limit its use during routine shopping situations [10, 11].

Given these demands on consumers, many countries have introduced front-of-pack labelling (FOPL) schemes that present simplified nutritional information on the front of food packages, complementing the more detailed back-of-pack formats. The primary objective of these systems is to enable consumers to assess and compare the nutritional quality of products more quickly and easily during shopping [12, 13]. FOPL schemes differ in their degree of interpretability. Descriptive labels, such as reference intake information, provide numerical values without evaluating overall nutritional quality, whereas interpretative labels summarize the healthfulness of a product using visual cues such as colours or symbols [14]. International examples include the Health Star Rating [15], the Heart Symbol [16] and the Keyhole Logo [17].

One prominent interpretative FOPL scheme is the Nutri-Score, developed in France in 2014 and subsequently adopted in several European countries, including Germany [18]. The Nutri-Score summarizes the nutritional quality of foods using a five-level color-coded scale from A (dark green) to E (red) [19]. In Germany, the label was introduced on a voluntary basis in 2020 [20]. Surveys indicate high public acceptance: in one representative German survey, the Nutri-Score was rated as the most helpful and easiest-to-understand nutrition label among several alternatives [21]. Public awareness of the Nutri-Score in Germany rose from 44% in 2021 to 88% in 2024 [22]. Recent consumer research further illustrates this development. A 2025 nationwide consumer study found very high levels of recognition of the Nutri-Score in Germany (91%) [23].

Interpretative FOPL schemes such as the Nutri-Score are grounded in the assumption that consumers often lack the time, motivation, or nutritional literacy to evaluate detailed nutrient information at the point of purchase, and that simplified, salient cues can reduce this cognitive burden and support healthier purchasing decisions [24]. Unlike the NIP and the ingredients list, which provide detailed nutritional and compositional information that consumers can use to evaluate specific product characteristics, the Nutri-Score provides a simplified overall assessment of nutritional quality [25]. These approaches therefore serve complementary rather than interchangeable functions. Consequently, these three food labelling formats differ in the type of information they provide and the cognitive effort required to use them effectively [26], making it important to investigate their understanding and use together rather than in isolation.

By increasing the visibility and comparability of nutritional quality across products, FOPL schemes are intended to facilitate healthier purchasing decisions and may also encourage manufacturers to reformulate products, thereby improving the food supply [27–29]. However, evidence that these mechanisms translate into sustained improvements in purchasing behaviour, dietary quality, or nutrient-related outcomes remains inconsistent [30, 31].

Ultimately, whether these intended public health benefits can be achieved depends on consumers noticing, understanding and using food labels correctly [30]. Subjective understanding and use of the Nutri-Score, the NIP and the ingredients list can therefore be regarded as important preconditions for these labelling formats to achieve their intended effects. This is particularly relevant for parents, whose food purchasing decisions influence the dietary quality of the entire household, including their children [4].

In summary, Germany provides consumers with three key food labelling formats: the Nutri-Score, the NIP and the list of ingredients. While recent research has examined consumer perceptions of the Nutri-Score in detail, including large-scale consumer surveys in Germany [22, 32], little is known about how this front-of-pack label is used in conjunction with mandatory back-of-pack information such as the NIP and the ingredients list. Existing studies have typically focused on single formats in isolation. A comprehensive assessment of subjective understanding and use across multiple labelling formats is therefore still lacking.

The present study therefore pursued three specific aims: (1) to assess parents’ subjective understanding and use of the Nutri-Score, the NIP and the list of ingredients; (2) to identify characteristics associated with the likelihood of subjective understanding and/or using these formats; and (3) to explore reasons for non-use and evaluate parents’ perceptions of the labels. Understanding how these key food labelling formats are perceived and used among parents provides important insights for improving public health communication and optimizing food labelling policies.

## Methods

### Study design

The study was a cross-sectional online survey among adults living in households with children (in the following: parents).

### Study population

The study population comprised adults aged at least 18 years living in households with children. Separated parents with shared custody were able to participate if at least one child under the age of 18 years lived for at least 15 of 28 days in her or his household. The adult must have lived in Germany for at least one year, be responsible for at least 50% of the household’s food shopping and have sufficient knowledge of the German language to take part in a written online survey in German.

Quota sampling was used to approximate the distribution of households with at least one minor child in Germany with respect to net equivalent household income and municipality size.

### Recruitment

Recruitment and data collection took place between 20 February and 4 March 2024 via an online panel managed by Bilendi GmbH. Eligible participants received an email invitation containing a survey link to the Tivian/Unipark platform. Upon accessing the survey, they were provided with detailed information about the study and data protection and informed that participation was voluntary. Informed consent was obtained electronically by selecting the appropriate checkbox before proceeding to the survey. Screening and quota questions (net equivalent household income and municipality size, with quotas based on the 2023 microcensus [33]) were incorporated into the online questionnaire. If participants did not meet the inclusion criteria or if the quotas were full, they were redirected to the market research institute’s website with a relevant message.

To ensure response quality, three instructed-response items (attention checks) were embedded in the questionnaire (e.g., “Please select ‘rather agree’ for this statement.“). Participants who failed at least one of these items were also redirected to the market research institute’s website and excluded from the analyses.

### Data collection instrument

The data collection instrument was initially developed in the study “Knowledge and usage of nutrition-related preventive measures among adults of households at risk of poverty with children” (KENNER) of the “Multidisciplinary study of the health and nutritional status of persons living in households at risk of poverty with children in Germany” (MEGA_kids), which is described elsewhere [34]. In short, KENNER investigated the subjective understanding, use, reasons for non-use and evaluative aspects of the food-based dietary guidelines (FBDG), food labelling and diverse other nutrition-related preventive measures including mobile apps, school programmes, websites etc. among parents living in households at risk of poverty. All questions on food labelling (i.e., subjective understanding, use, reasons for non-use and evaluation of the Nutri-Score, NIP and ingredients list), as well as the corresponding response options, were identical to those used in KENNER. Compared with KENNER, the present survey additionally included one further nutrition-related preventive measure that is not part of the current analyses and one item assessing municipality size.

The KENNER questionnaire was designed to assess a broad range of nutrition-related preventive measures rather than food labelling specifically. The development of the KENNER questionnaire comprised (1) the systematic identification of nationally available nutrition-related preventive measures, (2) the operationalization of subjective understanding, use, reasons for non-use and evaluative aspects applicable across different preventive measures and (3) questionnaire development. As no validated questionnaire suitable for assessing the broad range of preventive measures included in the survey could be identified, study-specific items were developed. The operationalization was informed by an extensive literature review e.g. [35, 36], existing evaluation frameworks and an iterative multidisciplinary consensus process. No formal psychometric validation or pilot testing of the study-specific items was conducted. The evaluative dimensions were largely derived from the evaluation framework of the German National Action Plan IN FORM [35] and subsequently adapted to the characteristics of the respective preventive measure. The questionnaire was designed to assess participants’ subjective perceptions and experiences rather than objectively measured knowledge or behaviour.

Subjective understanding of the Nutri-Score, the NIP and the list of ingredients was assessed by the question “How well do you know …, if at all?” with answer options “I do not know it”, “I only know the name or the logo, but do not know what it is about”, “I know it and roughly know what it is about” and “I know it and know what it is all about”.

For analysis, the variable was dichotomized into no / low subjective understanding (combining ‘unknown’ and ‘know only the name or logo but do not know what it is about’) versus subjective understanding (combining ‘know it and roughly know what it is about’ and ‘know it and know what it is about’). This distinction follows the established separation between knowing a label and subjective understanding of what the labelling format represents [37, 38], as only the latter reflects a level of understanding that may enable consumers to use the information when making food-related decisions. Accordingly, the measure reflects participants’ subjective understanding rather than objectively assessed the understanding of the respective food labelling format [38, 39].

Participants reporting subjective understanding of the Nutri-Score, the NIP and/or the ingredients list were then asked whether they had ever used the respective food labelling format. Thus, the measure reflects self-reported experience with using the respective labelling format rather than regular or current use. If they reported subjective understanding of the respective food labelling format, but did not use it, they were asked why they did not use it (answer options: “I have no time”, “I have no interest”, “I do not know enough about it”, “I do not think it is of any use to me” and open ended “others”). Participants using the food labelling format were also asked about evaluative aspects including, for instance, a perceived increase in knowledge about (healthy) nutrition (e.g. “I learned something new about the nutrients in foods through the NIP.”), visibility (e.g. “The Nutri-Score is clearly visible on the foods I use.”), presentation (e.g. “The design of the Nutri-Score is clear and well-structured”) and comprehensibility of the information provided (e.g. “I can understand the information on a product’s ingredient list.”), suitability for everyday use (e.g. “Reading the ingredient list when shopping can be easily integrated into everyday life.”), motivation to eat healthy (e.g. “The NIP motivates me to choose healthy foods when shopping.”) and the implementation of a healthy diet through the information provided (e.g. “The NIP helps me to eat healthily”) with answer options “totally disagree”, “disagree a little” “neither disagree nor agree”, “agree a little”, totally agree” and “don’t know”.

Socio-demographics (e.g. age, sex) and health aspects (e.g. height, weight, chronic disease) were assessed by a slightly adapted version of questionnaires from the German health interview and examination survey for adults (DEGS) [40]. The body mass index (BMI) was calculated as the ratio between height and weight as follows: $\:\frac{kg}{{m}^{2}}$ where kg is the body mass in kilograms and m is the body length in meters. The classification of BMI into underweight, normal weight, overweight and obese was based on the thresholds defined by the World Health Organization (WHO) for adults aged 18 and older [41]. The net household equivalised income was calculated in line with the Federal Government’s Poverty and Wealth Report [42] and then broken down into three terciles. The level of education was based on the highest general and vocational qualification and was defined and categorized according to the International Standard Classification of Education (ISCED) as low (ISCED 1–2), medium (ISCED 3–4) and high (ISCED 5–8) [43].

Attitudes toward the effectiveness of a healthy diet were assessed using an adapted version of the German questionnaire “Einstellungen zu gesunder Ernährung” (EGE) developed by Diehl [44], for which no formal psychometric validation has been published. From the original instrument, four items reflecting attitudes towards the effectiveness of a healthy diet (e.g., “Eating healthily is an important determinant of a long and healthy life”, “A healthy diet has a positive impact on everyone’s health”) and four items reflecting perceived overload by nutritional information (e.g., “I am tired of listening to what I should eat or should not eat”, “If one believed everything that is said about nutrition today, one wouldn’t be allowed to eat or drink at all.”) were selected. A small number of items were adapted by simplifying the wording while retaining its original meaning (e.g. “A sensible dietary pattern has a beneficial effect on every person’s health” was adapted to “A healthy diet has a positive effect on everyone’s health”). The second dimension was labelled “perceived overload by nutritional information” to better reflect the content of the selected items. Responses were recorded on a five-point Likert scale ranging from strongly disagree to strongly agree. A higher mean score on the subscale assessing attitudes towards the effectiveness of a healthy diet indicates a more positive attitude towards the impact of a healthy diet on health, whereas a higher mean score on the subscale assessing overload by nutritional information indicates a greater perceived overload by nutrition-related information. The adapted subscales showed acceptable internal consistency, with Cronbach’s α values of 0.83 for attitudes towards the effectiveness of healthy diet and 0.74 for perceived overload by nutritional information.

### Statistical analysis

Data analyses were performed using IBM SPSS statistics (version 27). Descriptive statistics (i.e. mean, standard deviation, absolute and relative frequency) were used to describe the sample in terms of socio-demographics and health aspects. Absolute and relative frequencies were used to present subjective understanding, use, reasons for non-use and evaluative aspects of the three food label formats among the sample. Logistic regression analyses were performed to ascertain the effects of age, sex, educational level, income, nationality, chronic disease, BMI category, attitudes toward the effectiveness of a healthy diet and perceived overload by nutrition information on the likelihood of reporting subjective understanding the Nutri-Score (model 1), the NIP (model 2) and the list of ingredients (model 3) and on the likelihood of using the Nutri-Score (model 4), the NIP (model 5) and the list of ingredients (model 6). The assumptions of logistic regression were assessed by evaluating multicollinearity using variance inflation factors (VIFs) and model fit using the Hosmer–Lemeshow goodness-of-fit test. Logistic regression analyses of food-label use were restricted to participants reporting subjective understanding of the respective food-labelling format, as only these participants were asked about their use of the label. Differences in evaluative aspects between the three food labelling formats were analysed using generalized estimating equations (GEE) with a binary logistic link function to account for the correlated responses of participants who evaluated more than one labelling format. Overall differences were assessed using Wald χ² tests, followed by Bonferroni-adjusted pairwise comparisons where appropriate. A *p*-value of < 0.05 was considered significant.

## Results

A total of 3 334 individuals were invited to participate in the survey. Approximately 9% (*n* = 304) were excluded for not meeting the inclusion criteria and 1 123 (33.7%) were excluded due to full quotas, leading to 1 427 sample-neutral dropouts. Additionally, 28 individuals declined to participate. Moreover, 687 participants were excluded from the analyses because they answered at least one of the three embedded attention check items incorrectly. Ultimately, the responses of 978 participants were included in the analysis.

The socio-demographic and health characteristics of participants are shown by Table 1. The sample had a mean age of 38.82 ± 9.87 years. Most participants were female (57.3%), had a high educational level (50.7%) and an equivalized net income between 1 400 and 2 800 euros (54.0%). Only few participants had a non-German nationality (2.6%). Half of participants had normal weight (50.0%), while 47.0% of participants were overweight or obese. The majority had no chronic disease such as hypertension, diabetes mellitus or mental disease (64.7%). The mean on the attitudes toward effectiveness of a healthy diet scale was 4.13 ± 0.66 and on the perceived overload by nutrition information scale 3.28 ± 0.85.

**Table 1:** Socio-demographic and health characteristics of the sample (*n* = 978)

Characteristic	Participants *n* (%)
Age in years^1^	38.82 ± 9.87
Sex	
female	560 (57.3)
male	417 (42.6)
diverse	1 (0.1)
Educational level	
low	36 (3.7)
middle	446 (45.6)
high	496 (50.7)
Net equivalent income in euro	
< 1400	231 (23.6)
1 400–2 800	528 (54.0)
≥ 2800	219 (22.4)
Nationality	
German	953 (97.4)
other	25 (2.6)
BMI	
underweight	29 (3.0)
normal weight	489 (50.0)
overweight	314 (32.1)
obesity	146 (14.9)
Chronic disease	
no	633 (64.7)
yes	345 (35.3)
Attitudes towards effectiveness of a healthy diet^1^	4.13 ± 0.66
Overload by nutrition information^1^	3.28 ± 0.66

^1^Continuous variables are presented as mean ± standard deviation. Mean scores are based on a five-point Likert scale ranging from 1 (strongly disagree) to 5 (strongly agree). Higher scores indicate stronger agreement

Table 2 shows subjective understanding of the three food labelling formats among participating parents. In total 84.8%, 83.7% and 80.5% of parents reported subjective understanding of the Nutri-Score, NIP and the list of ingredients, respectively, indicating that they knew at least roughly what each food labelling format was about. Overall, 73.7% of parents (*n* = 721) reported subjective understanding of all three food labelling formats, whereas 8.3% of parents (*n* = 81) reported no subjective understanding of any of the formats.

**Table 2:** Subjective understanding of the food labelling formats (*n* = 978)

“How well do you know …, if at all?”	Nutri-Score *n* (%)	NIP *n* (%)	List of ingredients *n* (%)
“I do not know it”	70 (7.2)	89 (9.1)	113 (11.6)
“I only know the name or the logo”	78 (8.0)	71 (7.3)	75 (8.0)
“I know it and roughly know what it is about”	372 (38.0)	292 (29.9)	294 (30.1)
“I know it and know what it is all about”	458 (46.8)	526 (53.8)	493 (50.4)

*NIP* nutrient information panel

Table 3 shows the results of the logistic regression analyses modelling the likelihood of subjective understanding of the Nutri-Score (model 1), the NIP (model 2) and the list of ingredients (model 3). All logistic regression models were statistically significant (model 1: X^2^ (13, *N* = 877) = 39.13, *p* < 0.001; model 2: X^2^ (13, *N* = 877) = 41.76, *p* < 0.001; model 3: X^2^ (13, *N* = 877) = 36.84, *p* < 0.001). Model 1 explained 6.9%, model 2 7.1% and model 3 5.9% (Nagelkerke R^2^) of the variance in knowledge. The models correctly classified 85.0% (model 1), 84.0% (model 2) and 80.5% (model 3) of cases. Parents who reported higher scores on the perceived overload of nutrition information scale were less likely to report subjective understanding of the Nutri-Score (OR 0.76, *p* = 0.019), the NIP (OR 0.72, *p* = 0.002) and the list of ingredients (OR 0.81, *p* = 0.041). German nationality was positively associated with subjective understanding of the Nutri-Score (OR 3.83, *p* = 0.002) and the NIP (OR 3.38, *p* = 0.007), while greater belief in the effectiveness of a healthy diet was positively associated with subjective understanding of the Nutri-Score (OR 1.60, *p* < 0.001) and the list of ingredients (OR 1.40, *p* = 0.007). In contrast, older age and a male sex was negatively associated with subjective understanding of the NIP (OR 0.98, *p* = 0.019; OR 0.56, *p* = 0.002) and the list of ingredients (OR 0.98, *p* = 0.0140; OR 0.58, *p* = 0.002). Educational level, net income, chronic diseases and BMI category were not associated with subjective understanding of any of the three labelling formats.

**Table 3:** Logistic regression analyses of subjective understanding of food labels and list of ingredients and diverse variables (*n* = 977)

	Model 1: Nutri-Score		Model 2: NIP		Model 3: List of ingredients	
	OR (95% CI)	*p* ^2^	OR (95% CI)	*p* ^2^	OR (95% CI)	*p* ^2^
Age	0.99 (0.97–1.21)	0.359	**0.98 (0.96–1.00)**	**0.019**	**0.98 (0.97–1.00)**	**0.040**
Sex^1^						
Male	0.83 (0.57–1.21)	0.331	**0.56 (0.39–0.82)**	**0.002**	**0.58 (0.41–0.82)**	**0.002**
Female	Ref.		**Ref.**		**Ref.**	
Educational level						
Low	0.64 (0.26–1.58)	0.328	1.12 (0.40–3.18)	0.830	0.48 (0.21–1.08)	0.075
Middle	0.79 (0.53–1.20)	0.267	0.91 (0.61–1.36)	0.654	0.98 (0.68–1.43)	0.931
High	Ref.		Ref.		Ref.	
Net income in euro						
< 1400	0.86 (0.48–1.56)	0.624	1.12 (0.63–1.98)	0.700	1.14 (0.67–1.95)	0.626
1 400–2 800	1.06 (0.65–1.74)	0.818	1.32 (0.84–2.09)	0.232	1.26 (0.82–1.92)	0.289
≥ 2800	Ref.		Ref.		Ref.	
Nationality						
German	**3.83 (1.62–9.05)**	**0.002**	**3.38 (1.39–8.23)**	**0.007**	1.96 (0.80–1.92)	0.143
other	Ref.		Ref.		Ref.	
Chronic disease						
No	0.79 (0.52–1.18)	0.249	0.90 (0.61–1.33)	0.588	0.90 (0.62–1.29)	0.560
Yes	Ref.		Ref,		Ref.	
BMI categories						
Underweight	0.59 (0.19–1.78)	0.345	0.63 (0.22–1.77)	0.380	0.47 (0.18–1.22)	0.121
Normal weight	0.71 (0.41–1.25)	0.242	1.10 (0.65–1.85)	0.731	0.92 (0.56–1.51)	0.748
Overweight	0.93 (0.51–1.67)	0.797	1.33 (0.77–2.30)	0.305	1.16 (0.69–1.95)	0.586
Obesity	Ref.		Ref.		Ref.	
Attitudes towards effectiveness of a healthy diet	**1.60 (1.23–2.08)**	**> 0.001**	1.27 (0.97–1.65)	0.079	**1.40 (1.09–1.78)**	**0.007**
Overload by nutrition information	**0.76 (0.61–0.96)**	**0.019**	**0.72 (0.58–0.89)**	**0.002**	**0.81 (0.67–0.99)**	**0.041**

*NIP* nutrient information panel, *OR* Odds ratio, *CI* confidence interval, *p* *p*-value

^1^One participant with diverse sex was excluded from these analyses; ^2^ Boldface values indicate statistically significant associations (*p* < 0.05)

Overall, 70.3% of participants (*n* = 668) reported using the Nutri-Score, 74.2% of participants (*n* = 726) reported using the NIP and 70.6% participants (*n* = 690) reported using the list of ingredients. More than half of the parents (58.8%, *n* = 575) reported using all three sources of nutrition information, while 4.1% (*n* = 40) reported not using any of them.

Table 4 shows the results of the logistic regression analyses modelling the likelihood of using the Nutri-Score (model 4), the NIP (model 5) and the list of ingredients (model 6). Again, all three logistic regression models were statistically significant (model 4: X^2^ (13, *N* = 877) = 32.90, *p* = 0.002; model 5: X^2^ (13, *N* = 877) = 43.39, *p* < 0.001; model 6: X^2^ (13, *N* = 877) = 56.14, *p* < 0.001). Model 4 explained 6.5%, model 5 10.2% and model 6 13.2% (Nagelkerke R^2^) of the variance in usage. The models correctly classified 83.0% (model 4), 88.9% (model 5) and 87.9% (model 6) of cases.

**Table 4:** Logistic regression analyses of using food labels and the list of ingredients and diverse variables

	Model 4: Nutri-Score (*n* = 830)		Model 5: NIP (*n* = 818)		Model 6: List of ingredients (*n* = 786)	
	OR (95% CI)	*p* ^2^	OR (95% CI)	*p* ^2^	OR (95% CI)	*p* ^2^
Age	0.98 (0.96–1.00)	0.082	1.00 (0.98–1.03)	0.770	1.01 (0.98–1.03)	0.543
Sex^1^						
Male	1.16 (0.78–1.73)	0.457	**0.56 (0.35–0.90)**	**0.015**	**0.53 (0.33–0.85)**	**0.008**
Female	Ref.		Ref.		Ref.	
Educational level						
Low	0.60 (0.23–1.60)	0.306	1.09 (0.32–3.66)	0.895	2.40 (0.49–11.69)	0.280
Middle	1.14 (0.75–1.73)	0.531	1.36 (0.82–2.26)	0.241	**1.78 (1.06–2.98)**	**0.028**
High	Ref.		Ref.		Ref.	
Net income in euro						
< 1400	1.12 (0.62–2.04)	0.700	1.29 (0.60–2.77)	0.514	1.34 (0.62–2.91)	0.452
1 400–2 800	1.21 (0.75–1.93)	0.436	1.02 (0.58–1.79)	0.959	0.98 (0.56–1.72)	0.952
≥ 2800	Ref.		Ref.		Ref.	
Nationality						
German	0.36 (0.05–2.84)	0.335	0.00 (0.00)	0.998	0.45 (0.06–3.56)	0.447
other	Ref.				Ref.	
Chronic disease						
No	0.68 (0.45–1.04)	0.072	0.80 (0.48–1.33)	0.385	0.95 (0.58–1.57)	0.843
Yes	Ref.		Ref.		Ref.	
BMI categories						
Underweight	0.89 (0.27–2.99)	0.849	0.59 (0.11–3.04)	0.528	1.35 (0.16–11.37)	0.784
Normal weight	0.77 (0.43–1.36)	0.364	0.48 (0.22–1.06)	0.068	0.58 (0.28–1.18)	0.134
Overweight	1.15 (0.63–2.11)	0.656	0.90 (0.39–2.06)	0.802	1.23 (0.57–2.67)	0.597
Obesity	Ref.		Ref.		Ref.	
Attitudes towards effectiveness of a healthy diet	**1.45 (1.08–1.94)**	**0.012**	**2.14 (1.55–2.94)**	**< 0.001**	**2.27 (1.64–3.15)**	**< 0.001**
Overload by nutrition information	**0.67 (0.53–0.84)**	**0.001**	0.86 (0.65–1.12)	0.265	**0.73 (0.56–0.96)**	**0.025**

*NIP* nutrient information panel, *OR* Odds ratio, *p* *p*-value

^1^One participant with diverse sex was excluded from these analyses; ^2^ Boldface values indicate statistically significant associations (*p* < 0.05)

Parents who scored higher on the effectiveness of a healthy diet scale were more likely to use the Nutri-Score (OR 1.45, *p* = 0.012), the NIP (OR 2.14, *p* < 0.001) and the list of ingredients (OR 2.27, *p* < 0.001). A lower score on the perceived overload by nutrition information scale was negatively associated with using the Nutri-Score (OR 0.67, *p* = 0.001) and the list of ingredients (OR 0.73, *p* = 0.025). Compared to women, men were less likely to report using the NIP (OR 0.56, *p* = 0.015) and list of ingredients (OR 0.53, *p* = 0.008). There also was a positive association with a middle educational level (compared to high educational level) and using the list of ingredients (OR 1.78, *p* = 0.028). Age, net income, nationality, chronic disease and BMI category were not associated with the likelihood of using any of the three sources of information on food packages.

Table 5 shows the number of participants who affirmed different reasons for not using the food labelling format among participants reporting subjective understanding of the respective food label format (“I know it and roughly know what it is about” and “I know it and know what it is about”). The reason for not using the Nutri-Score, NIP and/or list of ingredients was affirmed by most participants to be a lack of interest (39.7%, 52.9% and 47.2% respectively). Nearly every third Nutri-Score-nonuser agreed that the Nutri-Score was not of any use to him/her (32.8%), while only 11.8% of NIP-nonusers and 20.8% list-nonusers agreed to this statement. Overall, 21.6% of parents who did not use the Nutri-Score, 19.1% of parents who did not use the NIP and 18.1% of parents who did not use the list of ingredients affirmed that they did not know enough about it. Having no time was the least often affirmed reason for not using the Nutri-Score (7.8%), the NIP (11.8%) or the list of ingredients (16.7%).

**Table 5:** Reasons for non-use of food labels (multiple responses)

Reason	Nutri-Score *n* (%) (*n* = 116^1^)	NIP *n* (%) (*n* = 68^1^)	List of ingredients *n* (%) (*n* = 72^1^)
“I have no time.”	9 (7.8)	8 (11.8)	12 (16.7)
“I have no interest.”	46 (39.7)	36 (52.9)	34 (47.2)
“I don’t know enough about it”.	25 (21.6)	13 (19.1)	13 (18.1)
“I don’t think it’s any use to me.”	38 (32.8)	8 (11.8)	15 (20.8)
Other reason	3 (2.6)	0 (0.0)	2 (2.8)

*NIP* nutrient information panel

^1^Reasons for non-use were only assessed among participants who reported subjective understanding of the label but not using it

Table 6 shows participants’ agreement with the evaluative aspects of the three food labelling formats. Overall, significant differences between the three labelling formats were observed for most evaluative aspects. No significant differences were found regarding perceived affordability or motivation to choose healthy foods when shopping.

**Table 6:** Evaluative aspects related to food labels

Evaluative aspects^1^	Nutri-Score *n* (%) (*n* = 688^2^)	NIP *n* (%) (*n* = 726^2^)	List of ingredients *n* (%) (*n* = 690^2^)	Test
I have learnt something new about the nutrients in food via the …	400 (58.1)^a^	478 (65.8)^b^	475 (68.8)^b^	Wald-χ²(2) = 21.61, *p* < 0.001
The … is clearly visible on the foods I use.	594 (86.3)^a^	534 (73.6)^b^	472 (68.4)^c^	Wald-χ²(2) = 277.81, *p* < 0.001
The design of the … is clear.	599 (87.1)^a^	534 (73.6)^b^	383 (55.5)^c^	Wald-χ²(2) = 209.53, *p* < 0.001
I understand the … information on a product.	462 (67.2)^a^	552 (76.0)^b^	461 (66.8)^a^	Wald-χ²(2) = 26.60, *p* < 0.001
The amount of information presented by … is just right for me.	387 (56.3)^a^	513 (70.7)^b^	396 (57.4)^a^	Wald-χ²(2) = 50.50, *p* < 0.001
I can afford products with a green/positive ….	497 (72.2)^a^	532 (73.3)^a^	491 (71.2)^a^	Wald-χ²(2) = 2.58, *p* = 0.28
I can use the … to quickly recognize whether food is healthy or not.	465 (67.6)^a^	488 (67.2)^a^	430 (62.3)^b^	Wald-χ²(2) = 7.42, *p* = 0.025
Reading/considering the … when shopping is easy to implement in everyday life	532 (77.3)^a^	455 (62.7)^b^	387 (56.1)^c^	Wald-χ²(2) = 85.09, *p* < 0.001
The … motivates me to choose healthy foods when shopping.	393 (57.1)^a^	443 (61.0)^a^	397 (57.5)^a^	Wald-χ²(2) = 6.00, *p* = 0.050
… helps me to eat healthily.	389 (56.5)^a^	449 (61.8)^b^	425 (61.6)^b^	Wald-χ²(2) = 8.02, *p* = 0.018

Percentages refer to the observed data. Overall differences between labels were tested using generalized estimating equations (GEE). The last column reports the Wald χ² test. Within each row, values with different superscript letters differ significantly according to Bonferroni-adjusted pairwise comparisons (*p* < 0.05)

*INP* nutrient information panel

^1^Number and percentage of participants who agreed a little or totally; ^2^Evaluative aspects were only assessed among participants who reported using the label

More than half of users of the Nutri-Score (58.1%), the NIP (65.8%) and the list of ingredients (68.8%) agreed that the respective label had increased their knowledge about nutrients in foods (overall *p* < 0.001). Pairwise comparisons showed significantly higher agreement for the NIP and the list of ingredients than for the Nutri-Score, whereas the difference between the NIP and the list of ingredients was not significant.

The Nutri-Score received the most favourable ratings for visibility (86.3%) and clarity of design (87.1%), followed by the NIP (73.6% and 73.6%) and the list of ingredients (68.4% and 55.5%, respectively) (both overall *p* < 0.001). All pairwise comparisons were statistically significant.

A higher proportion of participants agreed that they understood the information provided by the NIP (76.0%) than by the Nutri-Score (67.2%) or the list of ingredients (66.8%) (overall *p* < 0.001). Likewise, the amount of information was more frequently perceived as “just right” for the NIP (70.7%) than for the Nutri-Score (56.3%) or the list of ingredients (57.4%) (overall *p* < 0.001). In both cases, agreement was significantly higher for the NIP than for the other two formats, whereas the Nutri-Score and the list of ingredients did not differ significantly.

No significant differences were observed regarding the affordability of products associated with the different labelling formats (*p* = 0.28). By contrast, significantly more participants agreed that the Nutri-Score enabled them to recognize healthy foods quickly (67.6%) and that its use was easy to integrate into everyday shopping (77.3%) than the list of ingredients (62.3% and 56.1%, respectively), whereas the NIP showed intermediate ratings. Ease of implementation differed significantly between all three formats (overall *p* < 0.001).

Finally, around 60% of participants agreed that the Nutri-Score (57.1%), the NIP (61.0%) and the list of ingredients (57.5%) motivated them to choose healthier foods when shopping. Participants more frequently agreed that the NIP (61.8%) and the list of ingredients (61.6%) helped them eat more healthily than the Nutri-Score (56.5%) (overall *p* = 0.018).

## Discussion

The primary aim of this study was to examine parents’ subjective understanding and use of three food labelling formats in Germany: the Nutri-Score, the NIP and the list of ingredients. Overall, 84.8%, 83.7% and 80.5% of parents reported subjective understanding of the Nutri-Score, the NIP and the ingredients list, respectively. Although direct comparisons are limited by differences in the wording of the survey questions and sample characteristics (parents with predominantly German nationality and medium to high levels of education vs. overall population), the proportion of parents reporting subjective understanding of the Nutri-Score in the present study was higher than the proportions of German consumers who reported that they could imagine or knew what the Nutri-Score means in surveys conducted in 2021 (73.9%) [45] and early 2020 (70.3%) [46]. This pattern is consistent with increasing consumer exposure to the label and its growing presence in the retail environment over this period [47].

In the present study, 70.3% of parents reported using the Nutri-Score, again exceeding previous estimates [32, 48]. Differences in measurement approaches (self-reported ever use vs. frequency of use) and sample characteristics (parents with predominantly German nationality and medium to high levels of education vs. overall adult population) may partly explain these discrepancies. Research from France suggests that households with children are more likely to correctly interpret front-of-pack labels such as the Nutri-Score than households without children [49]. This is relevant from a public health perspective, as parental food purchasing decisions shape the home food environment and thereby influence children’s dietary intake and long-term health outcomes [4]. Evidence on whether parental label use translates into improved dietary outcomes for children remains limited. In a Canadian study, parental nutrition label use was associated with more favourable cardiovascular biomarkers among parents themselves, whereas a direct association with children’s cardiovascular biomarkers and adiposity was not observed [50]. Whether parental engagement with food labels such as the Nutri-Score, the NIP, or the ingredients list translates into measurable improvements in family-level dietary or health outcomes therefore remains an important question for future research.

More than half of the parents in the present sample (58.8%) reported ever having used all three labelling formats. While previous studies have rarely examined multiple labelling formats simultaneously, this finding is consistent with research indicating that many consumers combine different sources of nutrition information when making food choices [51].

The second aim of the study was to identify characteristics associated with subjective understanding and use of the different labelling formats. Consistent with previous research [52], men were less likely than women to report subjective understanding or use of the NIP and the ingredients list. However, the most consistent predictors across models were perceived information overload and attitudes toward the effectiveness of healthy eating.

Parents who reported feeling overwhelmed by nutrition information were less likely to report subjective understanding or use of several labelling formats. This finding is consistent with recent German consumer research indicating that many individuals perceive nutrition communication as complex and sometimes contradictory [23]. Although FOPL formats such as the Nutri-Score are intended to simplify nutrition information [12], they operate within a broader information environment characterized by competing health claims, media debates and evolving dietary recommendations [53]. Perceived information overload may be associated with reduced engagement with food labels, but it is also possible that both are influenced by common underlying factors. Future longitudinal studies should examine the direction of this association.

Conversely, stronger beliefs in the effectiveness of healthy eating were consistently associated with subjective understanding and self-reported use of food labels. One possible explanation is that individuals who perceive diet as an important determinant of health may be more inclined to seek and use nutrition information, including food labels. For example, a cross-sectional survey study among Israeli consumers found that the perceived importance of nutrition and healthy food choice was a significant predictor of the frequency of food label use [54].

Interestingly, no association was observed between label use and chronic disease status or BMI category. Although earlier reviews suggested greater label use among individuals with diagnosed health conditions [52], more recent evidence is mixed [55]. Taken together, these findings indicate that health status alone may not consistently be associated with self-reported label use. The reasons underlying these inconsistent findings remain unclear and warrant further investigation.

Although several characteristics were significantly associated with subjective understanding and use of the different labelling formats, the explanatory power of the regression models was limited. This suggests that additional factors not assessed in the present study, such as nutrition literacy [56], healthful eating self-efficacy [57] and subjective importance of health [58], may also contribute to consumers’ engagement with food labels.

The third aim concerned reasons for non-use and perceptions of the labels. Lack of interest was the most frequently reported reason for not using the Nutri-Score, the NIP, or the ingredients list. Notably, 32.8% of parents who did not use the Nutri-Score reported that the label was not useful to them, a substantially higher proportion than for the NIP (11.8%) or the ingredients list (20.8%). One potential explanation discussed in previous research is the limited availability of the Nutri-Score on food products [48]. In a recent German consumer survey, 32% of respondents criticised that the label is not displayed on all products, making direct comparisons between foods more difficult, while 79% considered mandatory implementation of the Nutri-Score on all processed foods to be very or rather meaningful [48].

Another frequently reported reason for non-use was insufficient knowledge. Between 18% and 22% of non-users across formats reported insufficient knowledge as a reason for non-use. Qualitative research from France similarly indicates that many consumers recognize the Nutri-Score but lack understanding of how it is calculated [59]. Such limited understanding has been suggested to reduce trust in the label and weaken its perceived credibility [59]. Among participants who reported using the respective labelling formats, evaluations were generally positive but differed between formats. In particular, the Nutri-Score was perceived as highly visible and clearly presented, consistent with previous qualitative research [59]. By contrast, significantly more participants agreed that the information provided by the NIP was understandable and that the amount of information was “just right” than for the Nutri-Score and the ingredients list. Likewise, a higher proportion of participants reported that the NIP and the ingredients list helped them to eat more healthily than the Nutri-Score. The comparatively lower perceived comprehensibility of the ingredient list may reflect its complexity, as readability analyses suggest that ingredient lists require relatively high reading levels [60], exceeding recommended standards for accessible health information [61].

These subjective evaluations should be interpreted in the context of findings from experimental research. Experimental evidence suggests that the Nutri-Score may improve consumers’ ability to evaluate the nutritional quality of foods more effectively than the NIP [26] or other FOPL formats [49]. Eye-tracking data further indicate that extensive attention to the NIP may even reduce accuracy in healthfulness assessments, possibly due to cognitive overload [26]. As the present study assessed only participants’ subjective perceptions of the labelling formats, it cannot determine whether participants who perceived one labelling format as more understandable would also perform better in objective measures of comprehension or food-related decision-making.

### Limitations

The present study is the first to investigate subjective understanding, use, reasons for non-use and evaluative aspects of the Nutri-Score, the NIP and the list of ingredients among a large quota-based sample of parents in Germany. Nonetheless, it is important to acknowledge certain limitations inherent to the survey methodology. The first one is that all data are self-reported and might be subject to social desirability and recall bias. Although the extent of this bias remains unclear, studies which assessed the NIP use objectively reported much lower levels of NIP use [38, 62] than that reported in the present study, suggesting that self-reported use may overestimate actual use.

In addition, the questions related to the Nutri-Score, NIP and list of ingredients were part of a large questionnaire asking about diverse nutrition-related preventive measures and were not specifically adapted to food labels. For instance, use of food labels was assessed by the items “Have you (…) ever used the … or are you currently using it?” without any context information (e.g. “when grocery shopping”) or time limit (e.g. “the last 30 days”). It was therefore not possible to determine whether participants were still using the labels at the time of the data survey or to identify the context in which they were used. The context might, however, differ between the label formats. For instance, a Polish study observed that FOPL such as the Nutri-Score were more frequently read while shopping, likely due to their prominence and ease of access [63]. Back-of-package labels (BOPL) labels such as the NIP were more commonly consulted at home, where consumers had more time to analyse the content [63], implicating the FOPL may have a greater influence on immediate purchase decisions, while BOP labels may support more in-depth, post-purchase dietary assessment or planning. Furthermore, the dichotomous assessment of use did not provide information on how frequently or consistently participants used the respective labelling format or whether the information actually influenced their food choices. Consequently, the present findings should be interpreted as reflecting self-reported experience with using the respective labelling format rather than actual food-labelling practices or the extent to which food labels influence purchasing decisions.

Although quota sampling was used to approximate the distribution of parents in Germany with respect to municipality size and household income, recruitment was based on a commercial online access panel. Consequently, parents without internet access or who do not participate in online panels may be underrepresented. Furthermore, only municipality size and household income were included in the quota sampling procedure. Other characteristics, such as educational level, were not quota-controlled and differed from the distribution observed in the German Microcensus [33]. Therefore, despite the quota-based sampling approach, the findings should be interpreted as representative of the sampled online population rather than fully representative of all parents in Germany.

Furthermore, the proportion of participants without German nationality was relatively small. Therefore, associations involving nationality should be interpreted with caution, as the statistical power to detect differences between nationality groups was limited.

Finally, due to the cross-sectional design of the study, any causal conclusions cannot be made.

## Conclusions

Most parents in this study reported subjective understanding and use of the Nutri-Score, the NIP and the ingredients list. Perceived information overload and attitudes toward the effectiveness of a healthy diet were the most consistent predictors of engagement, highlighting the importance of consumer-related factors alongside characteristics of the labelling formats themselves. The observed differences in users’ evaluations further indicate that consumers perceive distinct strengths and limitations across the three labelling formats.

Clear and transparent communication about the purpose and limitations of different labelling formats also remains essential. For example, consumers should understand that the Nutri-Score provides an evaluative summary of nutritional quality but does not replace detailed nutrient information [19]. At the same time, although the ingredients list is widely used, relatively little is known about how consumers interpret and apply this information in practice, particularly in combination with other labelling formats.

Future research should investigate how consumer-related factors and characteristics of different labelling formats jointly influence engagement with food labels and whether this engagement translates into objectively measured food choices and dietary outcomes.

## Data Availability

The datasets used and analyzed during the current study are available from the corresponding author on reasonable request.

## Abbreviations

BOPL: Back-of-package label
BMI: Body mass index
DALYs: Disability-adjusted life years
DEGS: “German health interview and examination survey for adults”
EU: European Union
FBDG: Food-based dietary guidelines
FOPL: Front-of-pack labelling
GEE: Generalized estimating equations
ISCED: International Standard Classification of Education
KENNER: “Knowledge and usage of nutrition-related preventive measures among adults of households at risk of poverty with children”
MEGA_kids: “Multidisciplinary study of the health and nutritional status of persons living in households at risk of poverty with children in Germany”
NCDs: Non-communicable diseases
NIP: Nutrition Information Panel
VIFs: Variance inflation factors
WHO: World Health Organization
